# Health utility after emergency medical admission: a cross-sectional survey

**DOI:** 10.1186/1477-7525-10-20

**Published:** 2012-02-03

**Authors:** Steve W Goodacre, Richard W Wilson, Mike Bradburn, Martina Santarelli, Jon P Nicholl

**Affiliations:** 1 School of Health and Related Research (ScHARR), University of Sheffield, Sheffield, UK

**Keywords:** health utility, emergency medicine, hospital admission

## Abstract

**Objectives:**

Health utility combines health related quality of life and mortality to produce a generic outcome measure reflecting both morbidity and mortality. It has not been widely used as an outcome measure in evaluations of emergency care and little is known about the feasibility of measurement, typical values obtained or baseline factors that predict health utility. We aimed to measure health utility after emergency medical admission, to compare health utility to age, gender and regional population norms, and identify independent predictors of health utility.

**Methods:**

We selected 5760 patients across three hospitals who were admitted to hospital by ambulance as a medical emergency. The EQ-5D questionnaire was mailed to all who were still alive 30 days after admission. Health utility was estimated by applying tariff values to the EQ-5D responses or imputing a value of zero for those who had died. Multivariable analysis was used to identify independent predictors of health utility at 30 days.

**Results:**

Responses were received from 2488 (47.7%) patients, while 541 (9.4%) had died. Most respondents reported some or severe problems with each aspect of health. Mean health utility was 0.49 (standard deviation 0.35) in survivors and 0.45 (0.36) including non-survivors. Some 75% had health utility below their expected value (mean loss 0.32, 95% confidence interval 0.31 to 0.33) and 11% had health utility below zero (worse than death). On multivariable modelling, reduced health utility was associated with increased age and lower GCS, varied according to ICD10 code and was lower among females, patients with recent hospital admission, steroid therapy, or history of chronic respiratory disease, malignancy, diabetes or epilepsy.

**Conclusions:**

Health utility can be measured after emergency medical admission, although responder bias may be significant. Health utility after emergency medical admission is poor compared to population norms. We have identified independent predictors or health utility that need to be measured and taken into account in non-randomized evaluations of emergency care.

## Background

Patient outcomes need to be measured after emergency medical care for research, quality improvement and benchmarking of performance [1]. Mortality is widely used as an outcome measure in research, and risk-adjusted mortality can be used to compare systems of emergency care and drive quality improvement [2,3]. Health related quality of life, by contrast, is less commonly used as an outcome measure in emergency medicine research and has rarely been used in quality improvement [4-7]. However, some important emergency interventions, such as thrombolysis for stroke [8], affect health related quality of life rather than mortality. The impact of these interventions will only be identified if we measure health related quality of life.

Health related quality of life has been measured after hospital admission for major trauma [9-11] and specific illnesses, such as myocardial infarction [12] and stroke [13]. Major trauma is only responsible for a small proportion of emergency hospital admissions. Most admissions are for medical conditions, with patients increasingly presenting with mixed pathologies and multiple co-morbidities. We need to measure health related quality of life in the general emergency medical population if we are to estimate the effect of interventions and changes in service delivery upon the whole relevant population.

If both mortality and health related quality of life are measured then these can be combined to provide an overall measure of health, known as health utility. This measure allows comparison of outcomes between a wide range of different conditions and interventions affecting both mortality and morbidity. Combining mortality and health related quality of life in a single measure also overcomes a problem inherent in measuring quality of life alone, the "healthy survivor effect", whereby an apparent improvement in health related quality of life may be caused by an increase in mortality among patients with lower quality of life. However, health utility has not been widely used as an outcome measure in evaluations of emergency care and little is known about the feasibility of measurement, typical values obtained or baseline factors that predict health utility.

Health utility among emergency medical patients will clearly be influenced by many factors, especially pre-existing co-morbidities, and emergency treatment will be only one factor influencing outcome. Baseline measurement of health related quality of life (i.e. prior to emergency care) is subject to substantial logistical barriers and likely to be unfeasible or impractical for most evaluations, so any non-randomized evaluation of emergency care using health utility as an outcome needs to measure and take into account factors that predict health utility after emergency care. We therefore need to know which covariates predict health utility after emergency medical care, as well as knowing whether measurement is feasible, before health utility can become a widely-used outcome measure for emergency care.

This study was undertaken as part of the DAVROS study (Development And Validation of Risk-adjusted Outcomes for Systems of emergency care) and aimed to evaluate the use of health utility as an outcome measure in emergency care. Our specific objectives were to compare health utility of the population to region, age and gender adjusted normal values and to identify independent predictors of health utility. We did not aim to compare services in this evaluation or draw inferences about the effect of emergency health care upon health utility.

## Methods

We undertook a cross-sectional survey to measure health utility among patients recently admitted to hospital with a medical emergency using the EQ-5D self-complete questionnaire. We valued health utility using the EQ-5D for survivors and attributed a value of zero to those who had died by 30 days. The EQ-5D was developed following a review of existing generic health measures and consists of 5 questions relating to health status over the previous day [14,15]. The responses to the 5 questions allow patients to be classified into one of 243 possible health states. These health states were valued using preferences derived from the piloting of the questionnaire to produce a tariff for each state [15-17]. The tariff values overall health on a scale in which zero equates to death and one equates to perfect health. Negative values (health states worse than death) are possible.

The study took place in three emergency departments in Sheffield, Barnsley and Rotherham in South Yorkshire in the United Kingdom and in the Yorkshire Ambulance Service. These three emergency departments provide adult emergency services to a largely urban population of around 1 million. Patients were identified by review of hospital computer systems and selected if they were (a) alive and not in cardiac arrest when attended by an emergency ambulance, and (b) were then either admitted to hospital or died in the ambulance or emergency department. We excluded children (aged < 18 years), women with obstetric emergencies, adults with primarily mental health emergencies and injured adults aged under 65. We felt that these patients would have markedly different health utility from the majority of emergency medical admissions and/or would present particular difficulties in measurement. The threshold of 65 years for injuries was chosen as a crude means of including those with injuries likely to be secondary to or associated with medical complaints, while excluding those with primarily traumatic reasons for admission.

Hospital computer records and local Coroner's Office lists of deaths were checked 30 days after patient admission and any patient not recorded as being dead was sent a letter from the emergency department inviting them to take part in the research, along with an information sheet, consent form and copy of the EQ-5D questionnaire. If they were willing to participate they signed the consent form, completed the EQ-5D and mailed both to the University of Sheffield in a postage-paid envelope. They were asked to return the uncompleted questionnaire if they did not wish to participate. A single reminder was mailed two weeks after the initial mailing to non-responders.

Emergency department data, including patient age, gender, physiology (heart rate, respiratory rate, blood pressure, peripheral oxygen saturation and Glasgow Coma Score (GCS)), recorded co-morbidities and hospital admission within the previous 30 days, were abstracted from computer and paper hospital records by a researcher. Ambulance physiology data were recorded by paramedics on the standard patient report forms and then scanned onto an electronic database. Ambulance data were then matched to emergency department data using the ambulance dispatch code. Wherever possible the first physiological recording (i.e. the ambulance recording) was used. Where no physiology was recorded in the ambulance or the cases could not be matched to the patient report form the emergency department physiology data were used. Each patient had an International Classification of Diseases version 10 (ICD-10) code attributed by hospital clerical staff as part of routine management. All data were entered onto a secure online database managed by the University of Sheffield Clinical Trials Unit.

Chi-square tests were used to test the association between baseline patient characteristics (age group, gender, ICD-10 code, hospital admission within the previous 30 days, recorded co-morbidities and hospital attended) and questionnaire response rate. Patients who had died by 30 days were attributed health utility of zero. Patients who had died effectively had a response rate of 100% whereas those who survived had a lower response rate. To account for this differential rate of missing data we upweighted the EQ-5D scores of questionnaire respondents by the inverse of the age- and sex- specific response rate. All analyses used the weighted scores. Health utility data for the normal United Kingdom population were used to calculate a regional, age and gender adjusted expected normal value for each patient [17]. Analysis of variance was used to test the association between baseline patient characteristics and health utility, and also between physiological variables and health utility. Finally multivariable linear regression was used to determine independent predictors of health utility. Continuous covariates were categorised for the purpose of displaying univariate associations and assessing linearity, but were included as continuous in the multivariable model. Functional form was assessed using fractional polynomials [18]. Missing data was handled by performing multiple imputation in which the candidate explanatory factors previously described were imputed on the basis of each other using chained imputation [19]. Reported results are based on multiple imputation results and the presented model is the averaged results from 50 imputations. All analyses were undertaken using Stat version 11 (Stata Statistical Software: Release 11. College Station, TX: StataCorp LP. 2009).

The study protocol was approved by the Leeds East Research Ethics Committee (reference 07/Q1206/24).

## Results

We identified 2427 eligible cases between 11 February 2008 and 5 May 2008 in Sheffield, 1673 cases between 19 November 2007 and 24 February 2008 in Barnsley and 1660 cases between 19 November 2007 and 25 February 2008 in Rotherham. Out of the total of 5760 cases, 541 (9.4%) were identified as having died by 30 days after attendance: 519 were identified from the hospital computer system with no additional cases from local Coroner's records, while 22 were identified after relatives returned the questionnaire or contacted us following inadvertent mailing of someone who had died. Completed questionnaires were received from 2488 patients (47.7% of those alive at 30 days), while 71 patients declined to participate by returning an empty questionnaire and the remainder did not respond.

Table 1 shows the proportion of patients who had died by 30 days after hospital attendance and the proportion of those mailed who responded, according to baseline patient characteristics. The response rate was lowest among the youngest and oldest, and highest in the 60-69 year group. Patients diagnosed with diseases of the circulatory system, musculoskeletal system or nervous system had higher response rates while those diagnosed with diseases of the skin and subcutaneous tissue, endocrine, nutritional and metabolic diseases, mental and behavioural disorders or neoplasms had lower response rates. Patients with a history of chronic respiratory disease or heart disease, and those on long-term steroid or warfarin therapy had higher response rates than those without these co-morbidities.

**Table 1:** Death and response rate in relation to patient characteristics

	N	Death rate n (%)	Response rate* n (%)	P-value for response rate*
Overall	5760	541 (9.4%)	2487 (47.7%)	
Gender				0.400
Females	2995	279 (9.3%)	1273 (46.9%)	
Males	2710	255 (9.4%)	1193 (48.6%)	
Age				< 0.001
Under 30	355	2 (0.6%)	108 (30.6%)	
30-39	368	4 (1.1%)	117 (32.1%)	
40-49	504	4 (0.8%)	200 (40.0%)	
50-59	516	19 (3.7%)	258 (51.9%)	
60-69	823	69 (8.4%)	440 (58.4%)	
70-79	1301	137 (10.5%)	636 (54.6%)	
80-89	1441	219 (15.2%)	594 (48.6%)	
90 or above	422	82 (19.4%)	122 (35.9%)	
Centre				< 0.001
Barnsley	1673	127 (7.6%)	779 (50.4%)	
Rotherham	1660	188 (11.3%)	733 (49.8%)	
Sheffield	2427	226 (9.3%)	975 (44.3%)	
Hospital admission within the last 30 days				0.007
Yes	879	93 (10.6%)	361 (45.9%)	
No	2734	259 (9.5%)	1138 (46.0%)	
Unknown	2147	189 (8.8%)	988 (50.5%)	
ICD-10 code**				< 0.001
Certain infectious and parasitic diseases	118	24 (20.3%)	47 (50.0%)	
Diseases of the circulatory system	1074	148 (13.8%)	506 (54.6%)	
Diseases of the digestive system	530	29 (5.5%)	228 (45.5%)	
Diseases of the genitourinary system	296	22 (7.4%)	114 (41.6%)	
Diseases of the musculoskeletal system and connective tissue	114	1 (0.9%)	59 (52.2%)	
Diseases of the nervous system	180	6 (3.3%)	94 (54.0%)	
Diseases of the respiratory system	1060	174 (16.4%)	451 (50.9%)	
Diseases of the skin and subcutaneous tissue	50	4 (8.0%)	12 (26.1%)	
Endocrine, nutritional and metabolic diseases	96	4 (4.2%)	32 (34.8%)	
Injury, poisoning and certain other consequences of external causes	845	47 (5.6%)	339 (42.5%)	
Mental and behavioural disorders	120	4 (3.3%)	42 (36.2%)	
Neoplasms	110	46 (41.8%)	23 (35.9%)	
Symptoms, signs and abnormal clinical and laboratory findings, not elsewhere classified	1088	24 (2.2%)	508 (47.7%)	
Other	60	2 (3.3%)	29 (50.0%)	
Unknown	19	6 (31.6%)	3 (23.1%)	
History of chronic respiratory disease	746	96 (12.9%)	359 (55.2%)	< 0.001
History of heart disease	1902	211 (11.1%)	898 (53.1%)	< 0.001
History of active malignancy	266	84 (31.6%)	86 (47.3%)	0.91
History of asthma	661	44 (6.7%)	291 (47.2%)	0.792
History of diabetes	896	79 (8.8%)	399 (48.8%)	0.464
History of epilepsy	235	18 (7.7%)	94 (43.3%)	0.192
On warfarin therapy	319	33 (10.3%)	180 (62.9%)	< 0.001
On steroid therapy	296	44 (14.9%)	146 (57.9%)	< 0.001

* excluding deaths within 30 days

**primary discharge diagnosis

Table 2 shows the responses to each EQ-5D question. Most respondents reported at least some problems with each aspect of health and only about one in four were able to perform their usual activities or free from pain or discomfort.

**Table 2:** Responses to individual EQ-5D questions

	No problems	Some problems	Severe problems
Mobility	701 (28%)	1682 (68%)	104 (4%)
Self care	1256 (50%)	1041 (42%)	190 (8%)
Usual activities	604 (24%)	1290 (52%)	593 (24%)
Pain/discomfort	552 (22%)	1517 (61%)	418 (17%)
Anxiety/depression	1147 (46%)	1116 (45%)	224 (9%)

**Figure 1:**
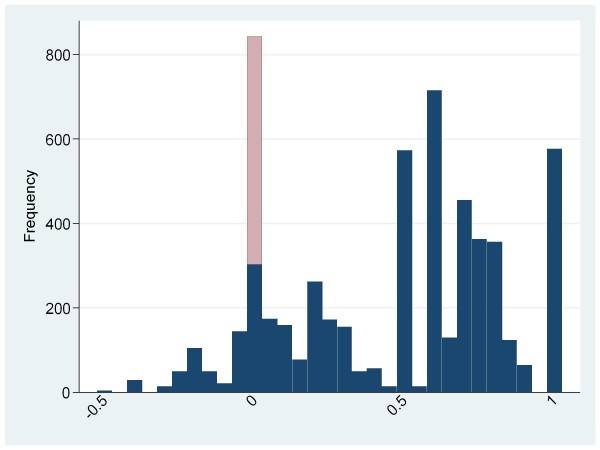
**Health utility of the study population**. Survivors are shaded in dark gray, dead patients in pale gray.

**Figure 2:**
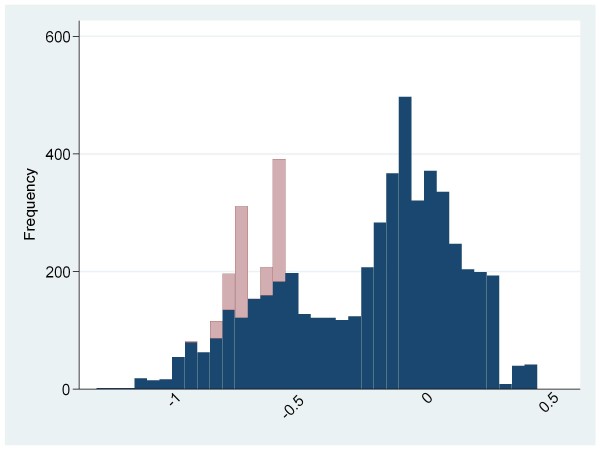
**Health utility loss compared to age and gender adjusted regional norms**. Survivors are shaded in dark gray, dead patients in pale gray.

Figure 1 shows health utility for the population and Figure 2 shows health utility compared to regional age and gender adjusted norms. Dead patients are included in these figures with a health utility of zero while values derived from the survey are weighted to allow for non-response. In both figures the dead patients are shaded in pale gray while the survivors are dark gray. Health utility was generally poor compared to age and gender adjusted regional norms. A substantial proportion of patients had health utility well below normal values (75% of patients had health utility below their normal value; mean loss 0.32, 95% CI 0.31 to 0.33) and a significant proportion (11%) had health utility below zero.

Mean health utility among survivors was 0.49 (standard deviation 0.35) while mean health utility in the whole cohort (i.e. including non-survivors) was 0.45 (0.36). Table 3 shows the association between patient characteristics and health utility. Mean health utility was lower in women, older patients and those with a recent hospital admission, recorded co-morbidities, neoplasms, diseases of the musculoskeletal system and connective tissue, diseases of the skin and subcutaneous tissue, diseases of the respiratory system, on steroid therapy and with abnormal physiological values or systolic blood pressure < 120 mmHg at presentation. On multivariable modelling (Table 4) health utility was reduced with increased age and lower GCS, varied according to ICD-10 and was lower among females, patients with recent hospital admission, on steroid therapy, or history of any of chronic respiratory disease, malignancy, diabetes and epilepsy. Other variables, most notably the physiological variables heart rate, respiratory rate, peripheral oxygen saturation and blood pressure, were not significant predictors of health utility in multivariate analysis.

**Table 3:** Health utility estimates stratified by patient characteristics

Factor	N	N dead	Mean	SD	Median	p
Overall	3028	541	0.45	0.36	0.52	
Gender						< 0.001
Females	1552	279	0.43	0.36	0.52	
Males	1448	255	0.48	0.37	0.59	
Age						< 0.001
Under 30	110	2	0.65	0.38	0.76	
30-39	121	4	0.58	0.37	0.69	
40-49	204	4	0.53	0.40	0.69	
50-59	277	19	0.47	0.36	0.59	
60-69	509	69	0.45	0.37	0.52	
70-79	773	137	0.43	0.35	0.52	
80-89	813	219	0.40	0.34	0.52	
90 or above	204	82	0.29	0.30	0.25	
Centre						0.071
Barnsley	906	127	0.47	0.36	0.59	
Rotherham	921	188	0.44	0.36	0.52	
Sheffield	1201	226	0.44	0.37	0.52	
Previous hospital admission						< 0.001
Yes	454	93	0.35	0.36	0.29	
No	1397	259	0.46	0.37	0.52	
Unknown	1177	189	0.48	0.36	0.59	
ICD-10 code						< 0.001
Certain infectious and parasitic diseases	71	24	0.43	0.41	0.52	
Diseases of the circulatory system	654	148	0.46	0.36	0.56	
Diseases of the digestive system	257	29	0.53	0.36	0.62	
Diseases of the genitourinary system	136	22	0.43	0.39	0.52	
Diseases of the musculoskeletal system and connective tissue	60	1	0.33	0.34	0.52	
Diseases of the nervous system	100	6	0.51	0.36	0.59	
Diseases of the respiratory system	625	174	0.39	0.37	0.38	
Diseases of the skin and subcutaneous tissue	16	4	0.36	0.38	0.52	
Endocrine, nutritional and metabolic diseases	36	4	0.63	0.34	0.69	
Injury, poisoning and certain other consequences of external causes	386	47	0.43	0.34	0.52	
Mental and behavioural disorders	46	4	0.46	0.36	0.52	
Neoplasms	69	46	0.18	0.33	0.00	
Symptoms, signs and abnormal clinical and laboratory findings, not elsewhere classified	532	24	0.50	0.35	0.59	
Other	31	2	0.55	0.35	0.59	
Unknown	9	6	0.41	0.47	0.19	
History of chronic respiratory disease	455	96	0.32	0.32	0.29	< 0.001
History of heart disease	1109	211	0.41	0.34	0.52	< 0.001
History of active malignancy	170	84	0.29	0.35	0.19	< 0.001
History of asthma	335	44	0.41	0.36	0.52	0.048
History of diabetes	478	79	0.40	0.35	0.52	0.003
History of epilepsy	112	18	0.39	0.38	0.52	0.074
On warfarin therapy	213	33	0.41	0.34	0.52	0.126
On steroid therapy	190	44	0.30	0.33	0.26	< 0.001
Glasgow coma scale						< 0.001
< = 8	111	67	0.24	0.36	0.03	
9-12	126	61	0.34	0.37	0.25	
13-15	2706	384	0.47	0.36	0.58	
Missing	85	29	0.32	0.33	0.36	
Oxygen saturation (%)						< 0.001
> 93(air)/> 98(O2)	1913	206	0.48	0.36	0.59	
90-93(air)/94-98(O2)	541	120	0.42	0.36	0.52	
< 90(air)/< 94(O2)	472	175	0.36	0.36	0.29	
Missing	102	40	0.35	0.37	0.36	
Systolic blood pressure (mmHg)						0.002
120-180	1967	274	0.46	0.36	0.58	
> 180	364	48	0.46	0.35	0.59	
100-119	365	104	0.42	0.37	0.52	
< 100	274	95	0.40	0.38	0.52	
Missing	58	20	0.31	0.36	0.36	
Heart rate (per minute)						0.003
60-100	1874	287	0.46	0.36	0.55	
101-130	745	153	0.42	0.37	0.52	
> 130	168	51	0.43	0.38	0.52	
< 60	188	33	0.51	0.37	0.62	
Missing	53	17	0.33	0.36	0.36	
Respiratory rate (per minute)						< 0.001
10-25	2359	329	0.48	0.36	0.59	
> 25	525	170	0.37	0.37	0.29	
< 10	13	5	0.25	0.38	0.08	
Missing	131	37	0.34	0.35	0.36	

Note: responders alive at 30 days are upweighted by the inverse of the age & gender specific response rate

**Table 4:** Multivariable analysis of factors associated with health utility (EQ-5D score)

	Beta	95% CI	p
Factor			
Gender (male v female)	0.049	0.016, 0.082	0.003
Previous hospital admission within 30 days (yes v no)	-0.101	-0.138, -0.064	< 0.001
ICD-10 code			
Certain infectious and parasitic diseases	(reference)		< 0.001
Diseases of the circulatory system	0.043	-0.066, 0.151	
Diseases of the digestive system	0.119	0.003, 0.235	
Diseases of the genitourinary system	-0.007	-0.133, 0.120	
Diseases of the musculoskeletal system and connective tissue	-0.091	-0.238, 0.057	
Diseases of the nervous system	0.127	-0.009, 0.263	
Diseases of the respiratory system	0.053	-0.056, 0.163	
Diseases of the skin and subcutaneous tissue	-0.275	-0.526, -0.023	
Endocrine, nutritional and metabolic diseases	0.180	0.011, 0.348	
Injury, poisoning and certain other consequences of external causes	-0.012	-0.123, 0.100	
Mental and behavioural disorders	-0.014	-0.165, 0.137	
Neoplasms	-0.091	-0.253, 0.072	
Symptoms, signs and abnormal clinical and laboratory findings, not elsewhere classified	0.085	-0.024, 0.194	
Other	0.128	-0.042, 0.298	
Unknown			
History of chronic respiratory disease	-0.094	-0.144, -0.043	< 0.001
History of active malignancy	-0.111	-0.190, -0.032	0.006
History of diabetes	-0.072	-0.118, -0.026	0.002
History of epilepsy	-0.101	-0.186, -0.016	0.021
On steroid therapy	-0.105	-0.169, -0.041	0.001
Age *			< 0.001
Glasgow coma scale *			< 0.001

* EQ5D was reduced with increased age and lower GCS; both were monotonic but non-linear decreases for which a quadratic model produced an adequate fit, Age: Fitted line is -0.0015*Age-0.000018*Age^2^, GCS: Fitted line is 0.0043*GCS +0.000852*GCS^2^

R-squared statistic for the model = 12.6%.

## Discussion

To our knowledge, this is the first study to measure health utility in an unselected cohort of patients after emergency medical admission. We found that health utility was markedly below regional age and gender adjusted norms and 11% of the population had health utility below zero. Measurement of mortality alone is inadequate in evaluating emergency medical treatments and services. If survivors have severely impaired health related quality of life then the value of interventions that improve survival alone may be open to question.

We identified patient characteristics that independently predicted health utility. Older patients, women, those with a low presenting GCS and those with recent hospital admission, on steroid therapy, or history of any of chronic respiratory disease, malignancy, diabetes or epilepsy had lower health utility. Non-randomized research and performance indicators measuring health utility should, if possible, measure and adjust for these covariates in analysis. Other baseline measures (heart rate, respiratory rate and blood pressure) were associated with health utility but were not independent predictors on multivariate analysis. These are potentially helpful findings because they indicate that the main independent predictors of health utility, with the exception of GCS, are likely to be routinely available and relatively easy to record for observational research. Additional efforts to record baseline physiology, with the exception of GCS, are unlikely to represent a worthwhile use of resources. However, it should be recognised that some of the factors were also associated with response rate. The estimated association between patient characteristics and health utility may therefore be influenced by responder bias. Furthermore, the R-squared statistic for the model (12.6%), whilst not especially low in the biomedical setting, suggests that the variables we identified only explain a modest proportion of variability. Examination of other co-morbidities, particularly those affecting mobility and mental health, could help to explain variability in health utility.

We deliberately selected a diverse sample. Emergency medical admissions include a substantial proportion of patients with ill-defined complaints or multiple morbidities, as reflected by the large group in our cohort with unclassified diagnoses. Including these patients is important to ensure a truly representative sample and attention is not focussed upon those with clearly defined single pathologies, but this can make it more difficult to draw inferences about the effects of intervention, if health utility data are used for this purpose.

Comparison of our data to previous studies of specific patient groups is complicated by differences in the timing of measurement, with most studies measuring health utility later than 30 days after admission, and previous studies limiting analysis to survivors. Even allowing for these differences our study suggests that at 30 days unselected emergency medical admissions, with a mean EQ-5D score among survivors of 0.49 (median 0.59), have worse health utility than patients suffering major trauma (median EQ-5D 0.73 at one year [9], mean 0.69 at 12-18 months [10]), stroke (mean 0.62 at 6 months [13]) or myocardial infarction (median 0.73 at 2 to 25 months [12]).

Our findings suggest that measuring health utility after emergency admission is feasible and potentially worthwhile. The response rate of 48% is sufficient for meaningful analysis and relatively high for an unsolicited mailing to an unselected sample, but carries a significant risk of responder bias. We have identified a number of factors that predict questionnaire response and need to be considered when planning future research. Although most deaths were identified though hospital systems we inadvertently mailed questionnaires to 22 patients who had died within 30 days. This risk was discussed with the ethics committee before the study and accepted as inevitable. However, inadvertent mailing to a deceased person has the potential to cause distress and should be taken into account when measuring health status.

An important implication of our study is that evaluations of emergency care, whether research or audit, that measure mortality without attempting to measure health related quality of life may fail to measure the true value of emergency care. Health utility after emergency admission is typically lower than age, gender and regional norms, and severely reduced in a substantial proportion.

Our study has a number of limitations. Only about half of the population responded and completed the questionnaire. We identified a number of factors predicting response which suggested that response was more likely among those aged 60-69 and those with co-morbidities, perhaps because these patients are more likely to be engaged in ongoing medical care. However, it is not clear what effect this bias will have on estimates of overall health utility.

The EQ-5D is a validated and widely used measure of health status, but when using the questionnaire patients do not directly value their health utility. Instead it is calculated using tariff values applied to the ratings they indicate on five dimensions of health. This means that the estimate of health utility generated by the EQ-5D for an individual may not equate to that individual's perception of their health status. This means that when the EQ-5D generates an estimate of less than zero for health utility we cannot assume that the individual rates their health status as being worse than death, only that their health status has been rated during EQ-5D validation as being worse than death. Furthermore, a single measurement at 30 days after hospital admission may represent a temporary state, associated with pain or loss of mobility for example, and should not be interpreted as a life not worth saving. Our study does not provide data on health changes after emergency medical admission or allow comparison to a control group, so we cannot draw inferences regarding the effectiveness or otherwise of the care provided.

The data presented in Figure 2 showing deviation from population norms should be interpreted with caution. The data are presented in this way to allow us to take into account population norms and should not be used to draw inferences about the quality of local emergency care or the implications of our findings for specific individuals. The cases with extreme values, in particular, may simply represent random variation with some people rating their health as good despite a low age and gender predicted value (and vice versa).

Finally, identifying patient characteristics that predict questionnaire response and health utility involved multiple hypothesis testing with the associated risk of spurious chance findings. We did not adjust statistical significance to account for multiple testing so would urge caution in interpreting findings with p-values between 0.05 and 0.01.

## Conclusion

Health utility can be measured after emergency medical admission, although responder bias may be significant. Health utility after emergency medical admission is poor compared to population norms. We have identified independent predictors or health utility that need to be measured and taken into account in non-randomized evaluations of emergency care. Further research is required to determine whether the findings of our study can be reproduced in other regions, countries and emergency care settings. Research is also required to identify reasons for non-response, to determine how responder bias may influence estimates of health utility and to explore methods to improve questionnaire response in this setting.

## Competing interests

The authors declare that they have no competing interests.

## Authors' contributions

SG and JN conceived and designed the study. RW and MS collected the data. MB analysed the data. SG wrote the first draft and all authors contributed to revision and preparation of the final draft. All authors read and approved the final manuscript.
